# Prognostic validity of the Periapical and Endodontic Status Scale for the radiographically assessed 2-year treatment outcomes in teeth with apical periodontitis: a prospective clinical study

**DOI:** 10.1186/s12903-021-01723-9

**Published:** 2021-07-19

**Authors:** Jelena Gudac, Kristina Hellén-Halme, Vita Machiulskiene

**Affiliations:** 1 Clinic of Dental and Oral Pathology, Lithuanian University of Health Sciences, Eiveniu str. 2, 50161 Kaunas, Lithuania; 2 Department of Oral and Maxillofacial Radiology, Faculty of Odontology, Malmö University, Carl Gustafs väg 34, 21421 Malmö, Sweden

**Keywords:** Cone-beam computed tomography, Apical periodontitis, PESS, Root canal treatment outcome

## Abstract

**Background:**

Endodontic treatment planning and treatment success evaluation are largely based on radiographic assessment of anatomical and treatment-related parameters of teeth with apical periodontitis (AP). This prospective clinical study aimed to assess radiographically the 2-year endodontic treatment outcomes for teeth with AP, and to evaluate prognostic validity of Periapical and Endodontic Status Scale (PESS).

**Methods:**

A total of 128 patients, representing 176 teeth with AP were examined by cone-beam computed tomography at baseline and at 24 months after endodontic treatment. Treatment outcome was evaluated using estimates of periapical radiolucency and the relationship between anatomical structures and location. The strength of the associations between these and treatment-related parameters was tested by logistic regression analysis. PESS sensitivity and specificity were calculated for every treatment risk group (low, moderate, high) of teeth.

**Results:**

One hundred and fifty-seven teeth, representing 350 root canals had a positive treatment outcome, while 19 teeth, representing 53 root canals had a negative treatment outcome at 24 months. The probability of negative outcome was 25 times higher in the moderate/high-risk group than in the mild-risk group of teeth (OR = 25.1; 95%CI [12.2–51.5]). Pre-treatment complications and retreatment cases with radiolucency were associated with negative outcomes (OR = 35.9; 95%CI [12.6–102.4]; OR = 26.437; 95%CI [10.9–64.1], respectively). PESS sensitivity and specificity was over 80% in all risk groups except for high risk group, due to very low number of cases.

**Conclusions:**

Endodontic treatment outcome depends on the severity of periapical changes. The presence of complications and retreatment cases with periapical lesions are associated with negative treatment outcome. The PESS is a valid instrument to predict outcome of teeth with low-moderate treatment risk of AP.

## Background

The success rates for root canal treatment reported in previous studies vary, depending on the methodological aspects as well as the evaluation criteria used [1, 2]. Moreover, various local pre-operative or, treatment-related factors (pre-operative periapical lesion size, presence of complications such as root canal blockage, crown or root perforation, root canal filling and coronal restoration quality, etc.), and possibly, general health conditions (presence of diabetes mellitus, steroid or, thyroxine therapies) can affect the treatment outcomes [2–4]. Most commonly, treatment evaluation is based on clinical findings (absence of clinical symptoms) and radiographic assessment of the periapical structures. Cone-beam computed tomography (CBCT) has become increasingly popular in endodontic practice because it allows three-dimensional imaging and provides better information about periapical changes as well as procedural errors and quality of root canal fillings, than digital periapical radiography [5–7]. However, the combined impact of different pre-operative and treatment-related factors on the treatment outcome is difficult to assess, as the magnitude of their effects varies at different time points after the treatment [3].

Various diagnostic indexes and guidelines using radiographic examination have been proposed to help clinicians evaluate the periapical tissue, treatment outcomes, and survival of root canal-treated or retreated teeth [8–10]. However, most of them lack complex evaluation of different diagnostic and treatment-related parameters. A well-known periapical index (PAI) developed by Orstavik et al. [11] classified periapical lesions into five severity levels according to reference radiographs of teeth with a confirmed histologic diagnosis. The PAI was based on two-dimensional radiographic evaluation of three-dimensional structures. Some years later, Estrela et al. [12] suggested a PAI based on CBCT to evaluate the extent of periapical lesions with additional variables, such as bone expansion or destruction. However, other important factors such as the number of affected roots, proximity of other anatomic structures (e.g., the sinus floor, nerve canals,) by the lesion, and lesion position (apical, on the side, or at the furcation) were not included.

The very ambitious proposal for clinical evaluation of teeth with respect to the restorative treatment need as well as their restorability was the Dental Practicality Index (DPI) [13]. The authors of this index aimed to include all possible determinants of the tooth function taking into account not only the local context (defined by the structural integrity, the periodontal and endodontic state), but also a number of other factors related to the patient’s oral and general health status. The validation of this index was based on the 4-year results regarding the effect of the coronal tooth structure loss on the clinical survival of root canal treated teeth [14]. However, the predictive validity of DPI with particular respect to the periapical healing was not elucidated.

Another recent development in this field is the Periapical and Endodontic Status Scale (PESS) developed by Venskutonis et al. [15]. In contrast to the DPI, the proposed PESS concentrated on the local risk factors and was based on a systematic evaluation of the radiographic diagnostic parameters related to the health status of the endodontically treated teeth. The diagnostic parameters of the PESS were selected from the well-known radiographic indexes suggested by Orstavik, Estrela and Eckerbom [11, 12, 16], and a large number of anatomical and treatment-related variables known to be associated with endodontic treatment outcomes were included. Thus, the CBCT-based assessment of anatomical variables such as presence and size of a periapical lesion, it’s topography, relationship with anatomic landmarks as well as coronal restoration quality, root canal filling length and homogeneity, treatment complications, and posts inside the canals served as the bases for scoring the periapical disease severity. The scale was developed to determine prognosis of the endodontic treatment outcome, with respect to three risk groups (low, moderate and high) defined by the periapical disease severity scores prior to treatment. However, the scale was based on a review of previous findings reported in the literature, and to our knowledge, still lacks clinical validation.

Thus, the aims of this prospective clinical study were to analyse the 2-year treatment outcomes in teeth treated endodontically for apical periodontitis, based on the CBCT-assessed periapical changes, and to evaluate the prognostic validity of PESS.

## Methods

### Participants

The study participants were adult patients who were admitted for general oral rehabilitation to Vilnius Implantology Center, Lithuania, and referred to a specialist for endodontic treatment during the period December 2016–December 2018.

A total of 140 patients, representing 200 teeth with AP were invited to participate in this prospective clinical study with 24 months of follow-up. Ten patients were excluded due to poor restorability of the teeth intended to treat (the remaining tooth structure less than 30%, impossible to achieve the adequate ferrule effect on the biomechanical performance of endodontically treated teeth [17]), and 2 patients declined to participate. Finally, 128 patients representing 176 teeth with AP were included in this study and were reminded by the office about the upcoming follow-up visits at 12 and at 24 months. All participants provided written informed consent. The study protocol was approved by the Ethical Committee of Biomedical Studies, Lithuania (Protocol No 111; 10.03.2016; edition No BE-2-27; 20.12.2016) and was carried out in accordance with relevant guidelines and regulations which were required and explained in the Ethical Committee approval and consent to participate form.

The patient inclusion criteria were as follows:

- No self-reported systemic diseases present
- At least one tooth with AP (necrotic pulp and/or filled root canals with radiographically determined signs of post-treatment endodontic disease requiring root canal retreatment)
- Absence of clinical periodontal inflammation on the tooth indicated for treatment, either anatomically intact or, with the reduced periodontium [18]
- Baseline CBCT images available prior to the study (images taken for general oral rehabilitation planning purposes no earlier than 1 month prior to endodontic procedures).

To allow using the standardised treatment protocol, pregnant women, immunosuppressed patients, and patients with symptoms of acute AP or presenting with un-restorable teeth (e.g., deep carious lesion, coronal cracks, root fracture) or with probing depths > 5 mm around the marginal bone were excluded from participation in the study.

Following diagnosis, a total of 176 teeth representing 403 root canals were subjected to endodontic treatment.

### Clinical and radiographic examinations

All participants were examined clinically and radiographically at baseline and at 24 months after endodontic treatment. An intermediate follow-up examination was carried out at 12-months; however, only the final study results are discussed in the present report.

The clinical examinations were performed by one examiner (JG) and included standard tests such as percussion, palpation, evaluation of the coronal seal, presence of sinus tracts, tooth mobility, and periodontal probing depth. For all diagnostic procedures, the patients were seated on a dental chair and a dental mirror and explorer (Dentsply Maillefer) were used. Case history (presence of clinical symptoms such as pain, swelling, abnormal bite, and time of previous treatment) was obtained from every patient.

Radiographic examinations of the patients were performed with CBCT imaging although the digital radiographs were taken as well, as part of the standardized root canal treatment and obturation protocol. In the present study, only CBCT data were included in the analysis. The baseline CBCT images (360—rotation) were already available after the general diagnostic and treatment plan proposed by the specialists in prosthetics and implantology. The follow-up CBCT images were obtained in 2 years after the endodontic treatment. Thus, in order to minimise radiation dose to the patients the 180-rotation of the respective area of interest (maxillary or, mandibular arch) was performed. All images were produced using an i-CAT scanner (Imaging Sciences International). The exposure parameters were as follows: 84 kV, 5 mA, 0.3 mm voxel resolution, 6 × 16 and 6 × 6 cm field of view, 18.3 s and 5 s acquisition time at baseline and at the final examination, respectively. The CBCT images were viewed as original i-CAT presentations (Apple) on a computer with a 27-inch flat panel display with a pixel resolution of 2,560 × 1,440 under dimmed ambient light less than 50 lux.

Radiographic assessment of the periapical status of the teeth (at baseline and after 24 months) was performed by the principle examiner (JG), using the evaluation criteria adapted from the PESS [15] (Table 1). Prior to evaluation of the CBCT images, an extensive training and calibration with an experienced radiologist (KHH) was performed. For the present study purpose, the intra-examiner agreement was assessed on the basis of double assessment of the CBCT radiographs from 30 randomly selected participants. The obtained results indicated very good agreement with regard to nearly all diagnostic parameters: the Cohen’s kappa values for examiner JG ranged from 0.84 to 1 [19]. To prevent the assessment bias, all the obtained CBCT images (128 baseline and 128 follow-up) were coded and mixed prior to the examination.

**Table 1:** Criteria for radiographic assessment of anatomical parameters adapted from the PESS [15]

Diagnostic parameters	Severity criteria
S, size of radiolucent lesion	S0: radiolucency does not exceed 2 times the width of the lateral PL^a^
	S1: diameter of well-defined radiolucency up to 3 mm
	S2: diameter of well-defined radiolucency 3–5 mm
	S3: diameter of well-defined radiolucency > 5 mm
R, radiolucent lesion in relation to the dental root	R1: radiolucency appears on one root
	R2: radiolucency appears on more than one root
	R3: radiolucency involves furcation area
D, location of bone destruction	D1: radiolucency around root apex
	D2: radiolucency is in contact with important anatomical structures
	D3: destruction of cortical bone

^a^Periodontal ligament

### Root canal treatment procedures

All procedures of root canal treatment or nonsurgical retreatment were carried out by a single endodontist (JG). The treatment was performed under local anaesthesia using 1.7 mL of 4% articaine hydrochloride containing epinephrine hydrochloride (1:1,000,000). The rubber dam system was applied for isolation. The root canals were prepared using sterilised, single-use endodontic Flexofiles, Pathfiles, and ProTaper nickel-titanium (NiTi) rotary instruments (Dentsply Maillefer) in a crown-down approach. Following the standardised treatment protocol, each canal was initially reamed with size 06, 08, and 10 stainless steel Flexofiles using the balanced force instrumentation technique to estimate the provisional working length, which was confirmed by an apex locator (Root ZX II; J Morita) to be 0.5 mm from the apex, as indicated electronically and verified radiographically. Then, the rotary Pathfiles and ProTaper instruments were used at speeds of 300 and 350 rpm, respectively, to prepare each root canal to at least an F1 master apical rotary file. The taper was tapered until the corresponding manual file fit snugly at the canal terminus.

Between instrumentations, the canals were gently irrigated with 2 mL 2.25% sodium hypochlorite (NaOCl) at 1 mm short of the working length using a 5-mL syringe and NaviTip 30-gauge tip (Ultradent), with 2–3 mm back-and-forth movements. For the final irrigation, 17% ethylenediaminetetraacetic acid (EDTA) (ENDO-Solution; PPH Cerkamed) solution was used for 1–2 min [20]. The irrigants, NaOCl and EDTA, were energised with a size 25 Endo-activator (Denstply Maillefer) for 1 min. The canals were then dried with paper points and filled with gutta-percha and AHPlus sealer (Denstply Maillefer) using a warm vertical compaction technique and gutta-percha points that had been disinfected with 2% chlorhexidine for 1 min [21]. The teeth were restored with permanent glass ionomer cores (GC Fuji IX) or with composite resin (Denstply Sirona) depending on the referring practitioner’s preference. A dental operating microscope (Carl Zeiss), with medium magnification of 8 ×, was used during the treatment procedures. Permanent restorations were performed within 1 month of root canal treatment.

### Study outcomes

The primary study outcomes were the transitions of the diagnostic parameters for each root estimated by comparing the baseline and the final CBCT images and indicating positive or negative treatment results after 2 years of follow-up.

A negative outcome was defined as increased/unchanged periapical radiolucency in relation to anatomical structures and/or location (PESS parameters S, size of radiolucent lesion, R, relationship between root and radiolucent lesion, and D, location of bone destruction) (Table 2).

**Table 2:** Treatment outcome definitions based on radiographically estimated transition events between baseline and final PESS scores of the periapical lesion size (S), it's relation with the root (R), and location of bone destruction (D)

Diagnostic parameters	Positive treatment outcome	Negative treatment outcome
Periapical lesion size, (S)	S3 → S2; S3 → S1; S3 → S0; S2 → S1; S2 → S0; S1 → S0;	S0 → S1; S0 → S2; S0 → S3; S1 → S2; S1 → S3;
	S1 → S1; S0 → S0	S2 → S2; S3 → S3
Periapical lesion relation with root, (R)	R3 → R2; R3 → R1; R3 → R0; R2 → R1; R2 → R0; R1 → R0; R1 → R1; R0 → R0	R0 → R1; R0 → R2; R0 → R3; R1 → R2; R1 → R3;
		R2 → R2; R3 → R3
Periapical lesion location, (D)	D3 → D2; D3 → D1; D3 → D0; D2 → D1; D2 → D0; D1 → D0; D1 → D1; D0 → D0	D0 → D1; D0 → D2; D0 → D3; D1 → D2; D1 → D3l;
		D2 → D2; D3 → D3

A positive outcome was defined as decreased periapical radiolucency, with respect to its size, in relation to anatomical structures and/or location (PESS parameters S, R, and D). Moreover, unchanged mild severity scores S1, D1, and R1 were regarded as positive outcomes as well (Table 2).

For multi-rooted teeth, the treatment outcome was determined using the root with the “worst” transition of the diagnostic parameters.

The secondary outcomes were the associations of the PESS diagnostic parameters with the negative treatment outcome expressed by odds ratios and 95% confidence intervals.

### Statistical analysis

Data analyses were performed using R version 3.6.3 software (R Core Team, R Foundation for Statistical Computing). Sample size calculation was based on the work of Patel et al*.* [22]. Thus, with a power of 0.9 and α = 0.05, a minimum of 150 teeth would be needed to assess the change of the periapical lesion size around the root by means of CBCT, over a period of 12 months. The 10% increase in the sample size to compensate for possible drop out would result in a total of 165 teeth. In the present study, 176 teeth diagnosed with AP were included to ensure maximum statistical power and precision.

Descriptive statistics were used to describe the distribution and changes in the diagnostic parameters S, R, and D of the treated teeth, over the 2-year period. Spearman’s rank correlation was used to estimate pairwise correlation between the anatomical parameters S, R, and D. The transition events as defined in Table 3 and the associations between different anatomical and treatment-related diagnostic parameters were analysed using the root canal as the unit of evaluation.

**Table 3:** Distribution of root canals (n = 403), with respect to transitions of the investigated parameters (S, R, and D) and treatment outcome

Diagnostic parameters	Positive treatment outcome, n			Negative treatment outcome, n		
	Changed to better	Remained unchanged	Total	Changed to worse	Remained unchanged	Total
Periapical lesion size, (S)	80	299	379	15	9	24
Periapical lesion relation with root, (R)	69	285	354	19	30	49
Periapical lesion location, (D)	67	319	386	15	2	17
Total*			350			53

^*^ “Total” denotes the transitions of the periapical status of the root canals based on all three parameters (S, R, and D), using the highest severity score to determine the treatment outcome. n, number of root canals

At baseline, three treatment risk levels were defined as follows. Thus, all baseline scores—0 or 1 on one or more of the S, R and D parameters were classified as being of mild risk. The moderate risk group comprised teeth with at least one score 2 on the S, R and D parameters. The teeth with at least one PESS score of 3 on S, R or D were assigned to the high-risk group.

In order to evaluate the prognostic validity of PESS, sensitivity and specificity values, as well as their confidence intervals were calculated for each treatment risk group. Thus, the scale sensitivity was calculated as the proportion of teeth with an AP-related periapical severity status that were well predicted by the PESS. The scale specificity was calculated as the proportion of teeth without an AP-related periapical severity status that were not predicted to have it, by the PESS.

Univariate logistic regression analysis was applied to estimate the association between the treatment risk groups and negative treatment outcome. For this purpose, the moderate- and high-risk groups were combined because of the very small number of root canals in high-risk group, and the low-risk group was used as the reference.

## Results

All 128 patients underwent the final examination 2 years after the endodontic treatment. The mean age of the participants was 46 years (range 18–70 years; SD 12.3). During the follow-up period, three teeth (2 upper first molars and 1 upper second incisor) of three patients were extracted due to vertical root fracture and they were considered as a negative treatment outcome. Apart from that, no patient had any symptoms at the final examination, and there were no complaints regarding mastication with the endodontically treated teeth throughout the study period. All coronal restorations remained intact 2 years after their placement.

**Fig. 1:**
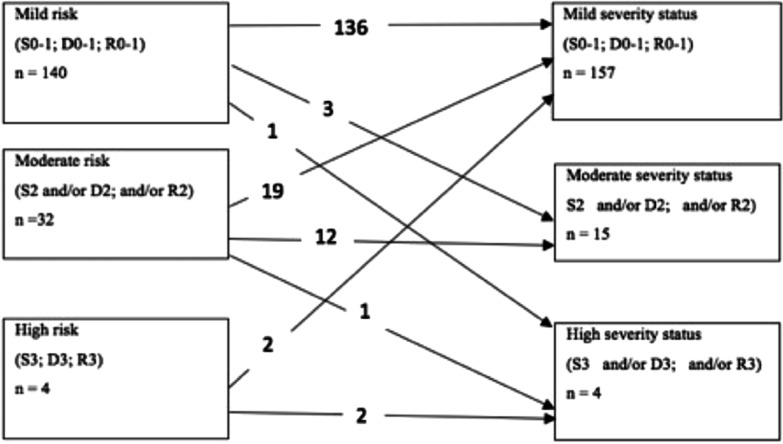
Distribution of teeth with AP to the risk groups at baseline, and, their periapical disease severity status after 2 years

Based on the CBCT images, after 2 years, 157 teeth showed positive treatment outcome, and 19 teeth, negative treatment outcome (89% and 11%, respectively, of the total number of teeth subjected to endodontic treatment). The baseline distribution of teeth according to the risk group (mild, moderate, or high) and their 2-year transitions with respect to the periapical status (based on the severity of S, R, and D parameters) are shown in Fig. 1. The distribution by tooth type, at baseline and after 2 years is presented in Table 4. This table shows that the molars were most prevalent in this study sample (48%), while the incisors/canines and the premolars comprised 24% and 28%, respectively, of the total number of teeth subjected to endodontic treatment. After 2 years, only 9% of the incisors/canines (4 teeth), 6% of the premolars (3teeth), and 14% of the molars (12 teeth) were recorded as having a negative outcome. The positive treatment outcome resulting from changing the status from “moderate” to “low” was recorded for 8 teeth of each type (Table 4). The increased counts of the high severity status were due to the extractions described above.

**Table 4:** Frequency distribution of teeth types by risk groups at baseline, and their periapical disease severity status after 2 years (yrs), (total n = 176)

	Low risk group		Moderate risk group		High risk group	
	Baseline	After 2 yrs	Baseline	After 2 yrs	Baseline	After 2 yrs
Incisors/Canines	38	39	2	2	3	2
Premolars	38	46	11	3	-	-
Molars	64	72	19	10	1	2
Total	140	157	32	15	4	4

The numbers of root canal transitions that occurred with every investigated parameter (size of radiolucency, S; radiolucency in relation to the root, R; location of bone destruction, D) are presented in Table 3. Thus, 87% (94%, 88%, and 96%, for S, R, and D, respectively) of the root canals showed positive treatment outcomes, while 13% (6%, 12%, and 4%, for S, R, and D, respectively) showed negative treatment outcomes after 2 years (Table 3). Moreover, there was a strong correlation between the selected study parameters (S, R, and D) (r = 0.96, *p* < 0.001).

The sensitivity and specificity values of the PESS for low, mild and high treatment risk groups are presented in Table 5. Thus, the PESS showed good specificity when assigning periapical lesion-free teeth to a low risk group, however, the sensitivity was lower, with the broad 95%CI limits. The scale showed good sensitivity (88%) when assigning teeth to medium risk group, although less specific. Finally, the PESS scale showed high sensitivity when assigning the teeth to high risk group. The specificity for high risk teeth was low due to the very low number of cases (Table 5).

**Table 5:** Validation of PESS capability to predict the treatment outcome of teeth with AP

Baseline treatment risk groups, n	Sensitivity, % (95%CI)	Specificity, % (95%CI)
Low risk, 140	79 (54–94)	87 (80–92)
Moderate risk, 32	88 (82–92)	80 (52–96)
High risk, 4	99 (96–100)	50 (68–93)

**Fig. 2:**
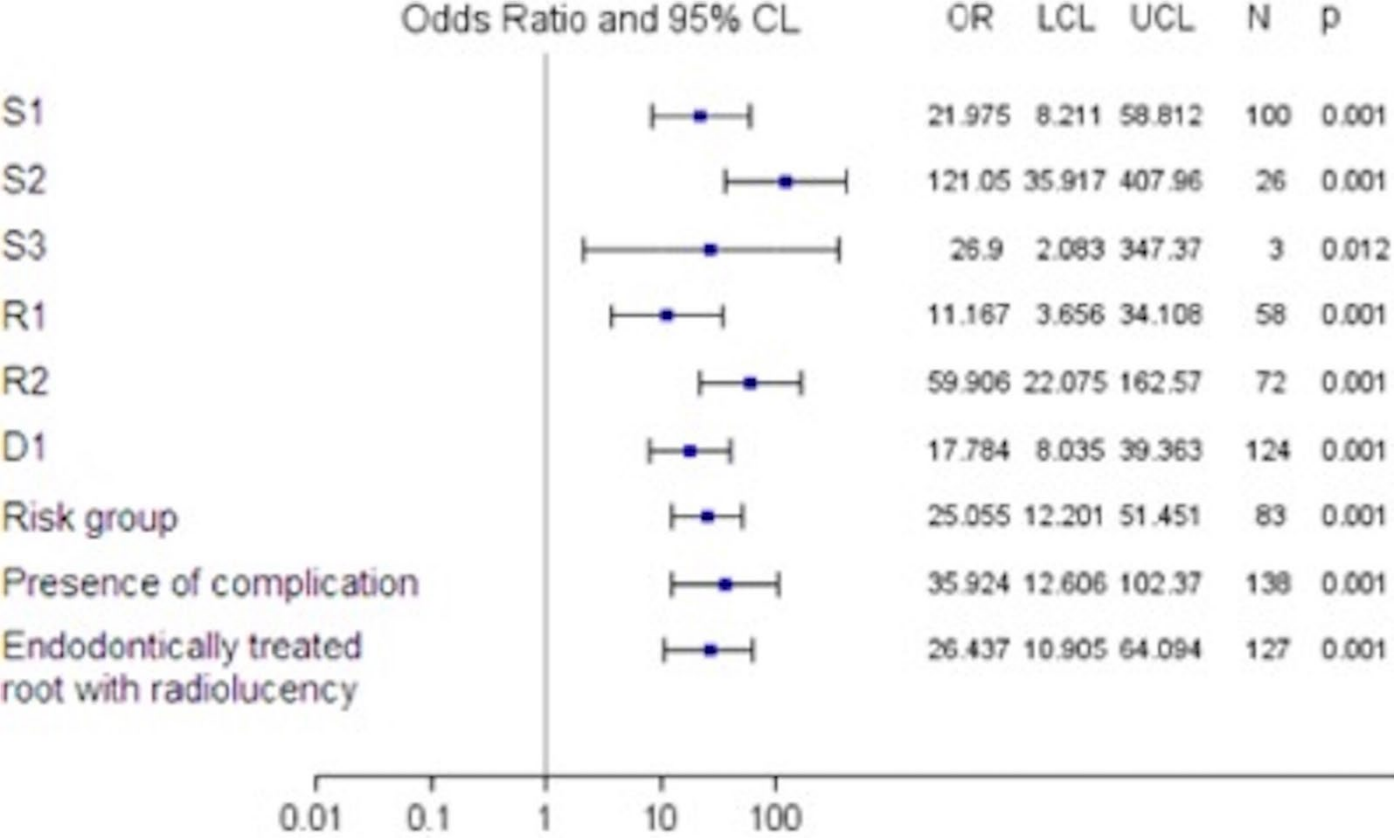
PESS parameters associated with the negative treatment outcomes of root canals

Univariate logistic regression analysis showed a 25-fold higher probability of negative treatment outcomes for the root canals attributed to moderate/high-risk groups prior to endodontic treatment (OR = 25.1; 95%CI (12.2–51.5), (Fig. 2). The series of logistic regression analyses showed a statistically significant association between negative treatment outcome and several treatment-related factors in the PESS. Thus, the presence of a complication prior to endodontic treatment (root perforation, root canal not treated/missed, root resorption, root/tooth fracture) and endodontic nonsurgical retreatment (endodontically treated root with radiolucency) were strongly associated with increased severity of the investigated parameters (S, R, and D) after 2 years (Fig. 2).

## Discussion

In this study, the long-term success of root canal treatment was analysed. The healing of apical periodontitis is a continuous time-taking process, and multiple host and treatment factors play a role in this course [23]. As suggested by several research groups, the minimal follow up period for evaluation of the endodontic treatment outcome should be at least, 12 months, however, there is still a significant healing potential during the second year of post-treatment period [24–26]. Thus, the 24 months follow up was carried out in the present investigation. Three major anatomical diagnostic parameters—size of radiolucency, radiolucency relation with the dental root, and location of bone destruction—were assessed on the CBCT images and served as the bases for evaluation of periapical healing. Of note, all three parameters strongly correlated with each other (r = 0.96, *p* < 0.001). Therefore, in clinical practice, it may be sufficient to use only one radiographic parameter of diagnosis, for example, size of radiolucency, without the risk of losing relevant information.

The root canal was used as a unit of analysis to investigate transitions of the diagnostic parameters (S, R, and D) over time and to test the associations between different anatomical and treatment-related diagnostic parameters. This decision was based on the assumption that using the tooth as a unit of analysis would allow the investigation of some of the root-level dependent variables such as size, relationship between the root and radiolucency, location of periapical radiolucency, root filling, presence of complications, etc. As shown by Hoskinson and co-authors [27], the proportion of teeth with successful treatment (77%) was similar to that of roots with successful treatment (75%), suggesting that the data obtained using the root as a unit of measure could be representative of treatment outcomes at the tooth level. The present study supported this conclusion as the proportion of teeth with successful treatment and the proportion of successfully treated roots differed by 2%.

To ensure the use of standardised treatment procedures, all root canals of the participating patients were treated by one experienced endodontist (JG), who strictly followed the treatment protocol. Overall, after 2 years of follow-up, positive treatment outcomes were observed in 89% of endodontically treated teeth. The most likely explanation of such an encouraging result would be the fact that the majority of baseline cases (140 teeth) were defined as “mild risk”, meaning that the radiographically detected periapical radiolucencies did not exceed 3 mm and involved no more than the apical area of one root in multi-rooted teeth. Moreover, nearly two-thirds of the teeth in the “moderate-risk” group showed a decrease in periapical severity status while the rest of this group (except for one tooth) remained within the same range of periapical severity (Fig. 1). Of the total of 19 teeth presenting with the negative treatment outcome, three were extracted during the follow-up period, due to vertical root fracture (VRF). They accounted for 2% of the negative treatment outcomes, in agreement with the findings of Olcay and co-authors [28]. Two of the extracted teeth belonged to the high-risk treatment group, while the third tooth had been assigned to the “moderate-risk” group at baseline. Of note, two extracted teeth had been restored with a glass post that is known to be a predisposing factor of VRF [29], while the third case was characterised as endodontic retreatment presenting with periapical radiolucency > 5 mm and separated instrument inside the canal. The main causes of VRF are still unclear. As suggested in the literature, a number of factors, such as root morphology and root canal anatomy, the amount of remaining dentin, age-related changes, and the degree of the removed dentine structure during root canal instrumentation or post-space preparation may play a role in inducing VRF [29, 30]. The very small number of treatment failures (such as VRF) in the present study does not allow us to draw any conclusions regarding the causative factors of root fractures; however, it supports the notion of the relevance of various predisposing factors related to treatment failure. The series of logistic regression analyses performed in this study revealed a statistically significant association between negative treatment outcome and several other treatment-related factors, as listed in the PESS. Thus, the presence of a pre-treatment complications (e.g., a procedural error, such as root perforation, a missed/untreated root canal, root resorption, instrument fracture inside the canal) and endodontically treated roots with radiolucencies were strongly associated with the increased severity of the investigated parameters (S, R, and D) after 2 years. This is not a new issue: an earlier systematic review by Ng and co-authors [2] reported a significantly higher probability of treatment success for nonsurgically retreated teeth without periapical lesions than for teeth with periapical lesions. Moreover, the success rates for nonsurgically retreated teeth had significantly reduced in the presence of procedural errors during primary endodontic treatment.

Previous studies on root canal treatment outcomes [4, 31] concluded that the treatment success rate was significantly influenced by the presence or absence of periapical pathology. These studies showed improved prognosis with endodontic treatment for small lesions, in agreement with the results of the present study.

An important aspect to be addressed when interpreting the present study results concerns the performance of CBCT: the baseline scans had been taken at 360-rotation (as part of the general treatment planning), while 180-rotation was performed at the final examination, in order to minimise radiation dose to the patients. This methodological inconsistency was considered acceptable based on the conclusions of several previous studies on artificial lesions where both 360° and 180° CBCT scans showed similar accuracy in the detection of periapical bone loss provided that all other exposure parameters were constant [32, 33]. Although the diagnostic accuracy is usually better for artificial lesions than for naturally occurring lesions, the high intra-examiner reliability of the principal examiner in the present study indicated the high quality of the analysed images. Moreover, this study did not compare the quality of the images. If any differences existed between first and second evolution according to how the investigation was made, we would expect fewer radiological findings in the second investigation, which was not the case.

A complex PESS index proposed by an international group of researchers some years ago [15] was designed to evaluate not only the status of periapical tissues but also the endodontic treatment quality. The authors included all pre- and postoperative factors potentially known to have an influence on the prognosis of endodontic treatment outcome. The diagnostic parameters included in PESS were gathered from previous scientific studies, and the diagnostic accuracy of the newly composed index was estimated using a retrospective study design. The intra- and inter-observer agreement scores varied from moderate to very good, leading the authors to the assumption that the PESS could be used universally in research and clinical practice.

The aim of this study was to validate the PESS in a prospective long-term clinical study. The diagnostic accuracy values were generally high in all risk groups. The scale demonstrated high potential to predict the reliable treatment outcome for the teeth assigned to the mild risk group (high specificity), with somewhat less reliable sensitivity to identify all mild-risk cases. On the contrary, the scale was most sensitive in assigning teeth to a moderate treatment risk group although the potential to predict the outcome was somewhat lower. Unfortunately, the very low number of teeth with high treatment risk precluded reliable evaluation of the predictive validity of the scale. The limitation of this study was that the majority of teeth subjected to endodontic treatment had been diagnosed with mild periapical changes, and the differentiation of moderate/high periapical disease severity was based on the limited number of cases. Another limitation of our study was the very small number of cases with complications of limited variety. An extended analysis of clinical cases with varying severities of periapical pathology using data from large multi-center clinical studies would be beneficial.

## Conclusions

This study highlighted that endodontic treatment outcome is related to the severity of the radiographically detected changes around the root. Moreover, the presence of complications and previous endodontic treatment (endodontically treated root with radiolucency) are associated with increased severity of periapical changes. Based on the present results, the modified PESS was found to be a valid instrument to predict a treatment outcome in clinical practice particularly in teeth with low and moderate treatment risk. The PESS can be used as an accessory tool with clinical evaluation in predicting the survival of teeth with apical periodontitis.

## Data Availability

The datasets used and/or analysed during the current study are available from the corresponding author on reasonable request.

## Abbreviations

CBCT: Cone-beam computed tomography
PAI: Periapical index
DPI: Dental Practicality Index
PESS: Periapical and Endodontic Status Scale
AP: Apical periodontitis
NiTi: Nickel-titanium
NaOCl: Sodium hypochlorite
EDTA: Ethylenediaminetetraacetic acid
OR: Odds ratio
VRF: Vertical root fracture
